# High frequency of subscapularis tears in rotator cuff repairs: a cross-sectional analysis of 302 cases

**DOI:** 10.1016/j.jseint.2025.09.008

**Published:** 2025-10-14

**Authors:** Saad M. AlQahtani, Victor A. Pacheco, Adeeba A. AlBadran

**Affiliations:** a Department of Orthopedic Surgery, College of Medicine, Imam Abdulrahman Bin Faisal University, Dammam, Saudi Arabia; b Orthopedic Department, Dr. Sulaiman Al Habib Medical Group – Al Khobar Branch, Al Khobar, Saudi Arabia; c Section of Orthopedic Surgery, Department of Surgery, King Abdullah Bin Abdulaziz University Hospital (KAAUH), Princess Nourah Bin Abdulrahman University, Riyadh, Saudi Arabia

**Keywords:** Subscapularis, Rotator cuff, Shoulder, Tendon tear, Cross-sectional study, Arthroscopy

## Abstract

**Background:**

Subscapularis tendon tears are frequently underdiagnosed in rotator cuff (RC) pathology. While prior estimates suggest around a 30% prevalence, newer data indicate this may be underestimated. This retrospective study evaluated the frequency and characteristics of subscapularis tears in patients undergoing arthroscopic repair for posterosuperior (PS) RC tears.

**Methods:**

A cross-sectional analysis was conducted on 302 patients who underwent arthroscopic repair for PS tendon tears (supraspinatus and/or infraspinatus) between March 2021 and April 2024. Subscapularis involvement was classified intraoperatively using the Lafosse system. Preoperative magnetic resonance imaging (MRI) accuracy was compared with surgical findings.

**Results:**

Intraoperative findings revealed subscapularis involvement in 61.9% of cases, significantly higher than traditionally reported (*P* = .004). Lafosse type II was the most common category (50.8%). Preoperative MRI missed 49.2% of subscapularis tears confirmed during surgery, with a positive predictive value of 100.0% and a negative predictive value of 55.6%.

**Conclusion:**

Subscapularis tears were present in nearly two-thirds of patients with PS RC pathology, suggesting the true prevalence is substantially underrecognized. These results underscore the diagnostic limitations of MRI and support the need for systematic intraoperative evaluation to ensure accurate identification and management of subscapularis involvement.

The subscapularis is the most robust and strongest muscle of the rotator cuff (RC); yet it has historically been underrecognized, labeled as the “*forgotten tendon.*”^10^^,^^11^ Subscapularis tears were first described in cadaveric studies by John Gregory Smith in 1834,^15^ and the first surgical repair was reported by Emil Hauser in 1954, who noted the rarity of the condition and that the tear could be only visualized after incising the anterior fascial sheath.^7^

Clinical interest in the subscapularis remerged in 1991, when Gerber and Krushell published outcomes following open repairs of isolated subscapularis tears.^6^ In 1994, Walch et al introduced the term *“hidden lesions”* after identifying subscapularis tears in 19 of 116 shoulders (16.4%), highlighting the diagnostic challenges associated with these injuries.^16^ Tear classification evolved in 2001 when Warner and colleagues coined the term “*anterosuperior tears*” to describe involvement of the superior subscapularis and anterior supraspinatus.^11^^,^^17^

Arthroscopic techniques advanced in the early 2000s, with Burkhart and Tehrany reporting the first series of arthroscopic subscapularis repairs in 2002,^3^ followed by the description of the “*comma sign*” in 2003 as a key arthroscopic landmark.^12^ Lafosse et al later introduced a widely adopted and comprehensive classification system in 2007, outlining surgical approaches based on tear type.^9^

In 2013, Denard and Burkhart published a detailed description of arthroscopic repair techniques along with clinical outcomes.^5^ Subsequent efforts to improve recognition and awareness have continued; notably, in 2019, Çetinkaya et al introduced the “*forelock sign*” (Fig. 1), a useful arthroscopic indicator of partial subscapularis tears.^4^

**Figure 1:**
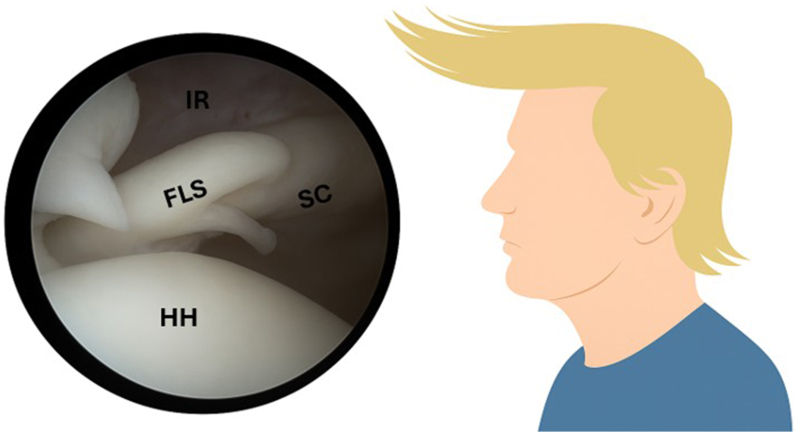
“The Forelock Sign.” Left shoulder, arthroscopic view from the posterior portal. IR, rotator interval; FLS, forelock sign; SC, subscapularis tendon; HH, humeral head. Source: Arthroscopic image obtained from a patient within the study cohort. *(Image © AlQahtani & Pacheco/Al Habib Medical Group)*.

This renewed focus has been supported by advancements in arthroscopic techniques and imaging, particularly magnetic resonance imaging (MRI), which have significantly improved early detection of subscapularis pathology, even in subtle or partial tears. Moreover, the identification of specific arthroscopic signs has contributed to more accurate intraoperative recognition of subscapularis involvement.

While isolated subscapularis tears are relatively uncommon, coexistence with posterosuperior (PS) RC tears (those involving the supraspinatus and/or infraspinatus) is frequently encountered in daily practice. Traditionally, this combined presentation has been reported and accepted around 30% of cases,^13^ but recent data suggest the prevalence may be substantially higher.^1^^,^^14^

This retrospective, cross-sectional study aimed to determine the frequency of combined subscapularis and PS tears among patients undergoing arthroscopic RC repair at the Orthopedic Department of Dr. Sulaiman Al Habib Hospital, Al Khobar, Saudi Arabia, from March 2021 to April 2024 inclusive. The hypothesis was that subscapularis involvement is common in RC pathology and that its frequency may have been historically under-reported due to limitations in detection and diagnostic emphasis.

## Materials and methods

This descriptive, retrospective cross-sectional study was conducted between March 2021 and April 2024 at the Orthopedic Department of Dr. Sulaiman Al Habib Medical Group (HMG), Al Khobar, Saudi Arabia. A total of 327 consecutive patients who underwent arthroscopic RC repair during the study period were initially identified. Of these, 25 patients were excluded due to incomplete operative reports or the absence of preoperative MRI in electronic medical records (EMR). The final analytic sample comprised 302 patients.

### Inclusion criteria

1. Patients aged 18 years and older
2. Signed surgical consent for RC repair
3. Primary RC repair
4. Availability of complete EMR, including preoperative MRI and intraoperative arthroscopic findings

### Exclusion criteria

1. Patients <18 years old
2. History of shoulder dislocation
3. Incomplete EMR or missing MRI/arthroscopic data
4. Diagnosis of frozen shoulder

### Radiologic assessment of subscapularis

Preoperative MRI scans were reviewed as part of the diagnostic evaluation. All MRI reports were initially provided by a senior radiology specialist and independently confirmed by a consultant radiologist with subspecialty expertise in musculoskeletal imaging. Any mention in the radiology report of terms such as “tendinosis,” “tendinitis,” “tear,” “partial tear,” or “full-thickness tear” was considered positive for subscapularis tendon injury. Reports indicating “intact tendon” or explicitly stating “no tear” were categorized as negative for subscapularis pathology. These criteria were applied to standardize the interpretation of preoperative MRI findings across the study population. If the report was not issued by the HMG radiology department or if the MRI was not available in EMR for assessment by the radiology department, the patient was excluded from the study.

### Subscapularis tear classification

Subscapularis tendon tears were classified intraoperatively according to the Lafosse classification, which categorizes tears into 5 types: Type I: partial tear of the superior one-third; type II: complete tear of superior one-third; type III: complete tear of the superior two-thirds; type IV: complete tear with centered head; type V: complete tear but eccentric head.^9^ The tear type recorded was determined by mutual agreement between the 2 attending shoulder surgeons at the conclusion of each procedure.

### Statistical analysis

**Figure 2:**
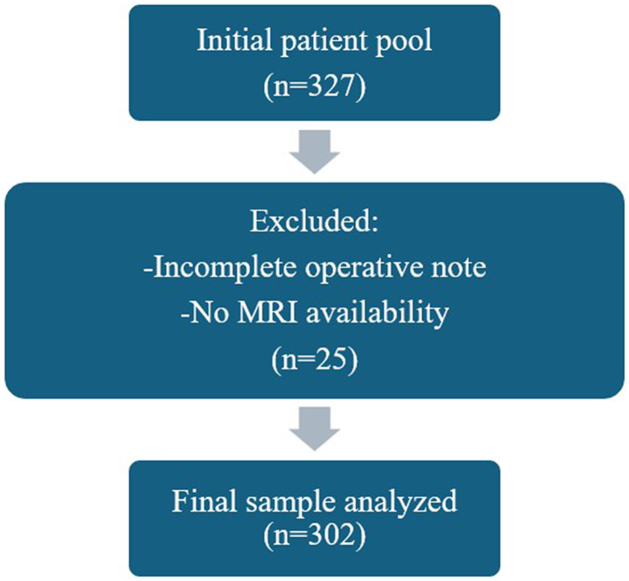
Sample selection flow diagram. *MRI*, magnetic resonance imaging.

Descriptive statistics were used to summarize patient demographics and injury patterns, including absolute and relative frequencies. Categorical variables were compared using the chi-square test, with statistical significance set at *P* < .05. The analysis was conducted with a 5% margin of error and a 95% confidence level. Positive predictive value (PPV) and negative predictive value (NPV) were calculated for MRI, using arthroscopic findings as the reference standard. All analyses were performed using IBM SPSS Statistics version 26 (IBM Corp., Armonk, NY, USA). Results are presented in tables and figures, including a flow diagram summarizing the patient selection process (Fig. 2). This study was approved by the institutional review board of the Research Center of Al HMG (IRB approval number: RC25.04.42).

## Results

**Figure 3:**
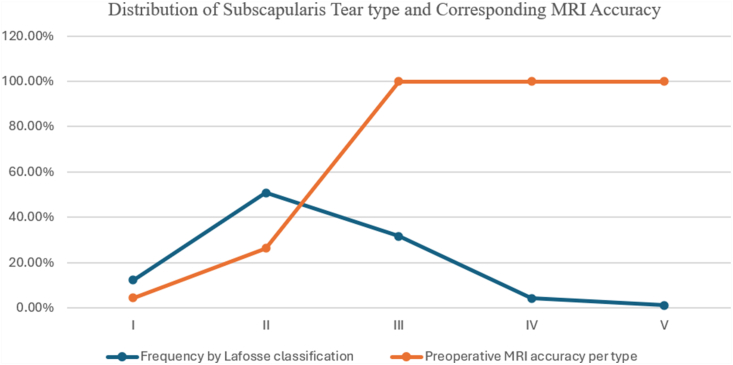
Distribution of subscapularis tears by Lafosse type and its corresponding percentage of preoperative MRI accuracy calculated per type among 187 patients undergoing arthroscopic rotator cuff repair. Source: data from study cohort. *MRI*, magnetic resonance imaging.

Intraoperative assessment revealed concomitant PS RC tears, with subscapularis involvement in 61.9% of cases and isolated PS RC tears in 38.1% (*P* = .004). Preoperative MRI detected 50.8% (95 of 187) of the subscapularis tears later confirmed intraoperatively, while missing 49.2% (92 of 187). The PPV was 100%, whereas the NPV was 55.6% (Table I). MRI demonstrated 100% diagnostic accuracy for subscapularis tears graded Lafosse type III and above, with missed diagnoses confined to type I (22 of 23) and type II (70 of 95) lesions (Fig. 3).

**Table I:** Comparative findings of subscapularis tears on MRI and surgery in patients undergoing rotator cuff repair.

MRI findings	Intraoperative: tear	Intraoperative:no tear	Total
MRI: tear	95 (31.5%)	0 (0%)	95 (31.5%)
MRI: no tear	92 (30.4%)	115 (38.1%)	207 (68.5%)
Total	187 (61.9%)	115 (38.1%)	302 (100%)

*MRI*, magnetic resonance imaging; *PPV*, Positive predictive value; *NPV*, negative predictive value.

*P* = .004.

PPV, 100.0%; NPV, 55.6%.

Source: data from study cohort.

Among the 187 patients with subscapularis tears confirmed intraoperatively, Lafosse type II lesions were most prevalent (n = 95, 50.8%), followed by types III (n = 59, 31.5%) and I (n = 23, 12.3%). More severe tear types IV (n = 8, 4.3%) and V (n = 2, 1.1%) were observed less frequently (Fig. 3). Of the 187 subscapularis tears, 168 were repaired using suture anchors, while 19 were treated with débridement. All tears managed with débridement were Lafosse type I. The choice between repair and débridement was made intraoperatively based on the extent of the tear and tendon quality, as determined by the operating surgeon.

## Discussion

The research revealed that 61.9% of patients with PS RC tears had concomitant subscapularis tendon involvement, a rate significantly higher than the widely accepted prevalence of approximately 30%.^13^^,^^14^ This underscores the importance of thoroughly evaluating the subscapularis tendon during surgical planning and intervention. Importantly, this distribution suggests that subscapularis lesions are more frequently present than absent in the setting of PS RC repair, supporting the study's central hypothesis that subscapularis involvement is common and may be underrecognized. The subscapularis plays a crucial role in glenohumeral joint stabilization, both statically and dynamically, contributing significantly to shoulder function. Our results are consistent with recent studies, such as Oeding et al in 2024,^14^^,^^15^ who reported a 60% prevalence of combined subscapularis and supraspinatus tears using machine learning models, and Adkinson et al in 2021,^1^ who found subscapularis tears in 60% of patients undergoing RC surgery.

When subclassifying subscapularis tears using the Lafosse classification, our study found type II lesions to be the most prevalent, followed by types III and I. More advanced tears, namely types IV and V, were notably less frequent. This distribution suggests a predominance of mild to moderate tears involving the superior portion of the tendon. Interestingly, while Adkinson et al^1^ reported type I as the most common, our findings indicate a higher prevalence of type II lesions, highlighting potential variations in tear patterns across different populations and surgeon interpretation.

The results suggest that the diagnostic accuracy of preoperative MRI is directly related to lesion severity. MRI demonstrates limited reliability in detecting partial-thickness tears, as reported by Brockmeyer et al, who found a sensitivity of only 51.6% and a specificity of 77.2% for such lesions.^2^ In our cohort, MRI identified only half of tears, emphasizing the need for improved preoperative diagnostic accuracy and MRI interpretation. Although MRI has a high PPV, its low NPV means a negative report does not reliably rule out a tear. This assertion aligns with the findings of Kilic et al, who demonstrated that both radiologists and surgeons frequently fail to detect subscapularis tears on preoperative MRI, highlighting the ongoing challenge in preoperative imaging interpretation.^8^ Collectively, this evidence emphasizes that MRI is less reliable for partial-thickness and low-grade tears but remains highly accurate in more advanced cases, reinforcing the importance of a multimodal diagnostic approach that integrates clinical evaluation, imaging, and intraoperative assessment to optimize surgical outcomes.

This study has certain limitations. As a cross-sectional design, it does not allow for causal inferences or temporal analysis of tendon injury progression. While all surgeries were performed by a single specialized shoulder surgery team, which reduces variability in intraoperative assessment, the interpretation of preoperative MRI was performed by different radiologists, potentially introducing inconsistency in imaging evaluation. Thirdly, only patients undergoing surgical repair were included, which may exclude less severe or asymptomatic tears and limit generalizability. Finally, the Lafosse classification has limited interrater reliability, which could partly explain differences in reported rates of concomitant subscapularis tears across studies.

## Conclusion

This study reveals a higher-than-expected prevalence of subscapularis tears in patients undergoing arthroscopic RC repair, often under-recognized on standard preoperative imaging. MRI sequences with the affected shoulder internally rotated and lightly flexed may improve detection of low-grade tears enhancing preoperative MRI accuracy; however, surgeons must be aware that intraoperative arthroscopic assessment is more sensitive than preoperative MRI and be prepared to repair any tear identified during surgery. Heightened awareness and systematic evaluation of the subscapularis can enable more targeted treatment and reduce persistent shoulder dysfunction.

## Disclaimers:

Funding: This research received no external funding.

Conflict of Interest: The authors, their immediate families, and any research foundation with which they are affiliated have not received any financial payments or other benefits from any commercial entity related to the subject of this article.
